# Anti-Amnesic Effect of *Agastache rugosa* on Scopolamine-Induced Memory Impairment in Mice

**DOI:** 10.3390/ph17091173

**Published:** 2024-09-05

**Authors:** Sohi Kang, Nari Lee, Bokyung Jung, Huiyeong Jeong, Changjong Moon, Sang-Ik Park, Seungpil Yun, Teresa Yim, Jung Min Oh, Jae-Won Kim, Ji Hoon Song, Sungwook Chae, Joong Sun Kim

**Affiliations:** 1 College of Veterinary Medicine and BK21 FOUR Program, Chonnam National University, Gwangju 61186, Republic of Korea; anakang@gnu.ac.kr (S.K.); bkjung@jnu.ac.kr (B.J.); hyyy0e@jnu.ac.kr (H.J.); moonc@jnu.ac.kr (C.M.); sipark@jnu.ac.kr (S.-I.P.); 2 Department of Anatomy and Convergence Medical Science, College of Medicine, Institute of Health Sciences, Gyeongsang National University, Jinju 52727, Republic of Korea; 3 Jeju Institute of Korean Medicine, Jeju-si 63309, Republic of Korea; nr4163@jikom.or.kr (N.L.); ojm4554@jikom.or.kr (J.M.O.); kjw8839@jikom.or.kr (J.-W.K.); jhsong@vtl.co.kr (J.H.S.); 4 Department of Pharmacology and Convergence Medical Science, College of Medicine, Institute of Health Sciences, Gyeongsang National University, Jinju 52727, Republic of Korea; spyun@gnu.ac.kr; 5 Global GreenFriends Co., Seocho-gu, Seoul 06569, Republic of Korea; 96trs@hanmail.net; 6 Vital to Life Co., Seongnam-si 13306, Republic of Korea; 7 Center for Companion Animal New Drug Development, Jeonbuk Branch, Korea Institute of Toxicology, Jeongeup 56212, Republic of Korea; 8 KMConvergence Research Division, Korea Institute of Oriental Medicine, 1672 Yuseongdae-ro, Yuseong-gu, Daejeon 34054, Republic of Korea

**Keywords:** *Agastache rugosa*, cognitive dysfunction, scopolamine hydrobromide, neuroprotection, cholinergic system dysfunction

## Abstract

*Agastache rugosa*, a traditional Asian herbal medicine, is primarily used for digestive problems; yet, its cognitive benefits remain unexplored. This study evaluated the anti-amnesic effects of *A. rugosa* extract (ARE) on scopolamine (SCO)-induced memory impairment in mice. Mice received 100 or 200 mg/kg ARE orally for 5 days, followed by SCO injection. The ARE demonstrated significant antioxidant (DPPH IC_50_: 75.3 µg/mL) and anti-inflammatory effects (NO reduction). Furthermore, the ARE significantly improved memory performance in the passive avoidance test (escape latency: 157.2 s vs. 536.9 s), the novel object recognition test (novel object preference: 47.6% vs. 66.3%) and the Morris water maze (time spent in the target quadrant: 30.0% vs. 45.1%). The ARE reduced hippocampal acetylcholinesterase activity (1.8-fold vs. 1.1-fold) while increasing choline acetyltransferase (0.4-fold vs. 1.0-fold) and muscarinic acetylcholine receptor subtype I (0.3-fold vs. 1.6-fold) expression. The ARE improved hippocampal neurogenesis via doublecortin- (0.4-fold vs. 1.1-fold) and KI-67-positive (6.3 vs. 12.0) cells. Therefore, the ARE exerts protective effects against cognitive decline through cholinergic system modulation and antioxidant activity, supporting its potential use as a cognitive enhancer.

## 1. Introduction

As human life expectancy increases, there is a corresponding rise in age-related neurodegenerative diseases. Consequently, many individuals suffer from dementia, which is becoming a huge economic burden on society [1]. Even in middle-aged adults, persistent stress, excessive alcohol consumption, insufficient sleep and depression may boost neuron fatigue and impair memory [2,3,4,5].

An important concept for cognitive impairment is cholinergic dysfunction, which states that impaired choline function blocks nerve signaling transmission and can result in learning and memory problems [6]. Acetylcholine (ACh) is a neurotransmitter that is found throughout the central nervous system and has a role in various brain functions, including the regulation of neuronal network synchronization and cognitive memory processes [7].

The overstimulation of acetylcholinesterase (AChE) can cause a decline in ACh levels, leading to the frequent prescription of AChE inhibitors (AChEIs), such as tacrine (TA), as useful drugs to treat cognitive impairments in dementia patients [8]. Nevertheless, the long-term use of AChEIs leads to an elevation in adverse effects, including cardiovascular and gastrointestinal side effects, because of the excessive stimulation of choline activity and muscarinic receptor activation [9,10]. Consequently, there is an increasing desire for the invention of pharmaceuticals that are effective and have minimal adverse effects to successfully manage cognitive impairment.

Oxidative stress is a significant factor contributing to memory loss [11]. The brain, which requires a substantial amount of oxygen, is particularly susceptible to oxidative damage [12]. Numerous preclinical and clinical studies have linked oxidative stress to memory loss. The production of reactive oxygen species (ROS) leads to oxidative stress, which, in turn, damages neurons [13]. Plant-derived polyphenols exhibit strong antioxidant properties, and research on the neuroprotective effects of natural products’ antioxidant properties has garnered significant attention from researchers [14,15,16].

*Agastache rugosa* Kuntze, also referred to as Korean mint, is a medicinal and functional herb cultivated in northeast Asian nations such as Korea, Japan and China [17]. It has been used as a traditional medicine to treat digestive problems, including nausea and vomiting [18], as well as diarrhea and abdominal pain [19]. *A. rugosa* contains different types of flavonoids. Tilianin (7-O-β-glucoside) and rosmarinic acid are the primary flavonoids in *A. rugosa* that have been demonstrated to enhance its therapeutic properties [20,21]. Its various pharmacological effects have also been identified, including antifungal, antiviral, anti-inflammatory, antioxidant and anticancer properties [22,23,24]. However, the potential preventive properties of *A. rugosa* against cognitive impairment have not been evaluated.

This study explored the pharmacological effects of *A. rugosa* on cognitive impairment and its underlying processes using a mouse model of scopolamine (SCO)-induced memory impairment.

## 2. Results

### 2.1. ARE Has Antioxidant and Anti-Inflammatory Activities

**Figure 1:**
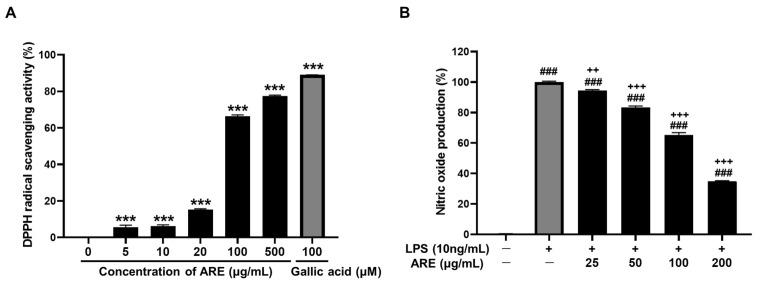
Effects of ARE on antioxidant activities. (**A**) DPPH radical scavenging assay; (**B**) NO scavenging activity. Data are presented as the mean ± standard error of three replicates (*n* = 3). *** *p* < 0.001 vs. CON group. ### *p* < 0.001 vs. LPS-non-treated group. ++ *p* < 0.01 and +++ *p* < 0.001 vs. LPS-treated group. CON, control; DPPH, 2,2-diphenyl-1-picryl hydrazyl hydrate; NO, nitric oxide; LPS, lipopolysaccharide; ARE, *Agastache rugosa* extract.

The antioxidant activity of ARE was evaluated by measuring its ability to scavenge the stable 2,2-diphenyl-1-picryl hydrazyl hydrate (DPPH) free radical (Figure 1A). The ARE (at concentrations of 5, 10, 20, 100 and 500 μg/mL) interacted with DPPH radicals. The radical scavenging activities of ARE demonstrated dose-dependent manners, with an inhibitory concentration (IC_50_) value of 75.3 μg/mL comparable to the positive control (gallic acid, IC_50_ = 8.8 µM) at a dose of 100 μM.

The anti-inflammatory action of ARE was evaluated based on its ability to scavenge nitric oxide (NO). In lipopolysaccharide (LPS)-stimulated RAW 264.7 cells, the production of NO increased significantly after exposure to LPS. However, treatment with ARE (25, 50, 100 and 200 μg/mL) significantly reduced the production of LPS-induced NO in a concentration-dependent manner (Figure 1B).

### 2.2. ARE Ameliorates Scopolamine-Induced Learning and Memory Impairments

#### 2.2.1. ARE Attenuates Scopolamine-Induced Impairment of Avoidance Memory

**Figure 2:**
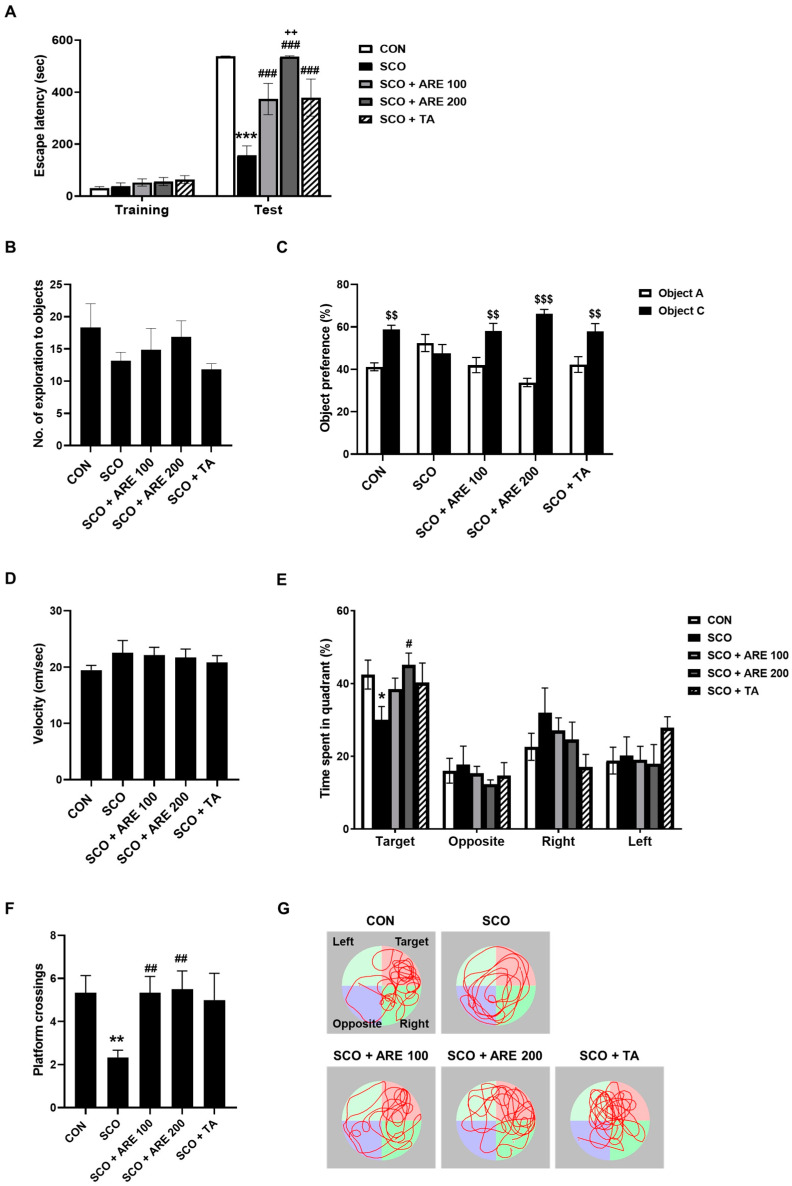
The effects of ARE on scopolamine-induced memory impairment. (**A**) ARE administration significantly increased latency time in the PA. In the NORM, all groups (**B**) showed equal preference for the two objects during training; however, following testing (24 h after training), all groups, except for the SCO group, (**C**) showed significant preference for the novel object. In the MWM, all groups (**D**) showed a similar swimming velocity during visible training; however, the administration of ARE significantly increased (**E**) the time spent in the target quadrant and (**F**) the number of platform crossings during the probe test. (**G**) Representative swimming paths during the probe test. Data are presented as the mean ± standard error (*n* = 6 per group). * *p* < 0.05, ** *p* < 0.01 and *** *p* < 0.001 vs. CON group. # *p* < 0.05, ## *p* < 0.01 and ### *p* < 0.001 vs. SCO group. ++ *p* < 0.01 vs. SCO + ARE 100 group. $$ *p* < 0.01 and $$$ *p* < 0.001 vs. Object A group. CON, control; SCO, scopolamine; ARE, *Agastache rugosa* extract; TA, tacrine; PA, passive avoidance test; NORM, novel object recognition memory test; MWM, Morris water maze test.

The effects of SCO and ARE on fear-related avoidance memory were assessed using the passive avoidance test (PA) (Figure 2A). During the training, there was no significant difference in the escape time between the different groups. However, during the post-24 h retention test, the PA indicated that SCO resulted in a decrease in the escape latency time compared to that of the control group (*p* < 0.001). However, the dose-dependent administration of ARE significantly prevented the decrease in escape latency time caused by SCO (*p* < 0.001). As a positive control, TA had effects similar to those of the ARE treatment (*p* < 0.001).

#### 2.2.2. ARE Attenuates Scopolamine-Induced Impairment of Novel Object Recognition Memory

The effects of SCO and ARE on novel object recognition memory were assessed using the novel object recognition memory test (NORM) (Figure 2B,C). During the training, there was no significant difference in the number of interactions with each object between the different groups. However, 24 h after the training sessions, the mice were evaluated using the NORM. The mice in the control group had a significant preference for the novel object (*p* < 0.01). In contrast, the mice treated with SCO showed a reduced preference (*p* = 0.8245). In addition, the administration of two doses of ARE significantly averted the reduction in recognition memory caused by SCO (*p* < 0.01). As a positive control, TA had effects similar to those of the ARE treatment (*p* < 0.01).

#### 2.2.3. ARE Reduces the Scopolamine-Induced Long-Term Spatial Memory Dysfunction

The effects of SCO and ARE on long-term spatial learning and memory were assessed using the Morris water maze test (MWM) (Figure 2D–F). In the visible platform training, there were no differences in swimming velocity, which meant the difference in alteration behavior was not due to locomotor activity (Figure 2D). In the probe test, the SCO group spent a lower percentage of time in the target quadrant (*p* < 0.05). However, the administration of 200 mg/kg of ARE significantly increased the percentage of time spent in the target quadrant (*p* < 0.05). SCO-treated mice also crossed the platform location fewer times than the control mice (*p* < 0.05). However, administering two doses of ARE significantly averted the reduced spatial memory caused by SCO (*p* < 0.05, Figure 2F). As a positive control, TA had effects similar to those of the ARE treatment (*p* = 0.1469, Figure 2E and *p* = 0.0643, Figure 2F).

### 2.3. ARE Modulates Scopolamine-Induced Alteration of Cholinergic Neurotransmitters

**Figure 3:**
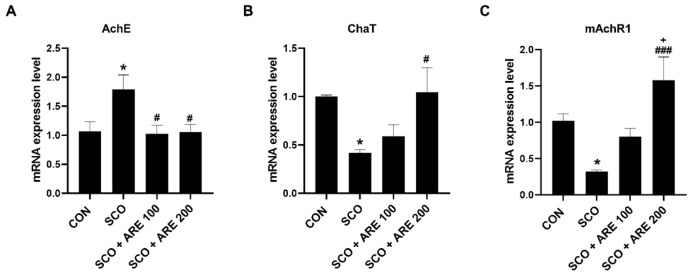
The effects of ARE on AChE, ChaT and mAChR1 mRNA expression. Bar graphs illustrating relative mRNA levels of (**A**) AChE, (**B**) ChaT and (**C**) mAChR1. Data are presented as the mean ± standard error (*n* = 6 per group). * *p* < 0.05 vs. CON group. # *p* < 0.05 and ### *p* < 0.001 vs. SCO group. + *p* < 0.05 vs. SCO + ARE 100 group. CON, control; SCO, scopolamine; ARE, *Agastache rugosa* extract; AChE, acetylcholinesterase; ChaT, choline acetyltransferase; mAChR1, muscarinic acetylcholine receptor subtype I.

The mRNA expression of AChE, ChaT and mAChR1 was detected using RT-qPCR. The graph bars show the changes in the hippocampal tissue after treatment with ARE. Figure 3A shows that after treatment, there was a significant decline in the AChE of the hippocampus in the group that received two doses of ARE compared to those observed in the SCO group (*p* < 0.05). Figure 3B demonstrates that the ChaT levels of the ARE 200 group were significantly higher than those of the SCO group (*p* < 0.05). Likewise, Figure 3C reveals a significant increase in the level of mAChR1 in the ARE 200 group (*p* < 0.001).

### 2.4. ARE Attenuates Scopolamine-Induced Impairment of Hippocampal Neurogenesis

**Figure 4:**
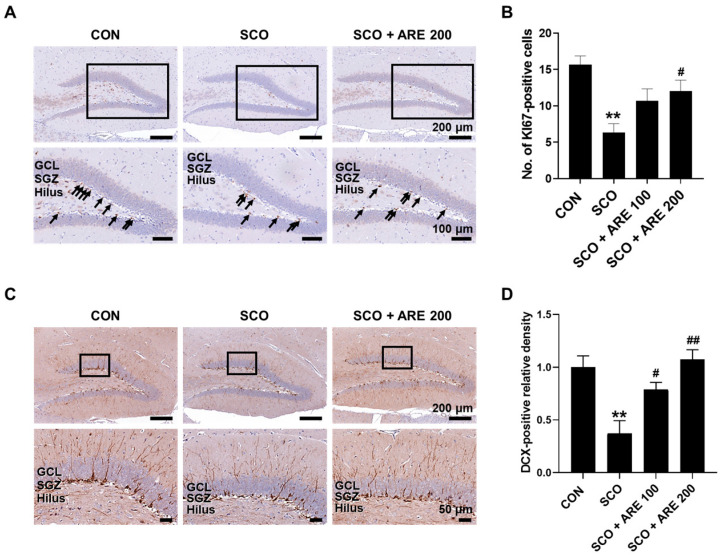
Immunohistochemical analysis of KI67 and DCX in the hippocampus of SCO-affected mice. (**A**) KI67-positive cells, which are associated with cell proliferation, were stained in the SGZ of the DG. (**B**) ARE administration significantly increased the number of KI67-positive cells in the hippocampus. (**C**) DCX-positive cells, which are associated with immature neurons, were stained in the SGZ of the DG. (**D**) ARE administration significantly increased the density of DCX-positive cells in the hippocampus. Data are presented as the mean ± standard error (*n* = 3 per group). ** *p* < 0.01 vs. CON group. # *p* < 0.05 and ## *p* < 0.01 vs. SCO group. Arrows indicate Ki67-positive cells. The square box is the location of SGZ in DG. DCX, doublecortin; CON, control; SCO, scopolamine; ARE, *Agastache rugosa* extract; GCL, granular cell layer; SGZ, subgranular zone; DG, dentate gyrus.

Immunohistochemical analysis demonstrated a significant decline in KI67-positive proliferative cells, which is indicative of a decline in neurogenesis in the hippocampus of SCO mice (Figure 4). Treatment with ARE 200 significantly increased KI67 immunoreactivity compared with the SCO-only treatment. Similarly, there was a significant decline in DCX-positive immature neurons, which is indicative of a decline in neurogenesis in the hippocampus of SCO mice. In addition, administering two doses of ARE significantly increased DCX immunoreactivity compared to the SCO-only treatment.

## 3. Discussion

The present study investigated the memory-enhancing benefits of ARE in a mouse model of cognitive impairment induced by cholinergic blockade. SCO, a blocker of muscarinic ACh receptors, is widely used in Alzheimer’s research [25]. It induces oxidative stress in rodents, interferes with neural choline pathways and impairs hippocampal function, leading to memory impairment [11,26]. Our results showed that the memory impairment induced by SCO observed in the PA, NORM and MWM was significantly improved with the administration of ARE. In addition, ARE regulated the production of cholinergic neurotransmitters and protected the adult neurogenesis stage. Furthermore, ARE effectively removed ROS, protecting the hippocampus against damage caused by SCO.

Behavioral assessments are essential for determining the effect of ARE on cognition. Three tests, including the PA, NORM and MWM, were conducted in this study. During the PA, ARE effectively reversed the reduced latency time caused by SCO, suggesting an enhancement in long-term memory [27]. To mitigate the stress induced by the fear-driven PA, the NORM approach was employed, which resulted in an improved object preference in the ARE-treated group, indicating an enhancement in recognition memory [28]. During the MWM, mice that received ARE treatment exhibited a longer duration in the target quadrant in the probe session, suggesting enhanced spatial memory [29]. We evaluated swimming speed to rule out the possibility of motor dysfunction due to SCO and found no alteration. These findings indicate that ARE significantly improves deficits in long-term recognition and spatial memory. Moreover, ARE showed a similar activity to TA, suggesting that ARE may have a potent anti-amnesic activity.

The cholinergic neurotransmitter system is a recognized mechanism that contributes to cognition and memory storage and its disruption leads to the development of Alzheimer’s disease [25]. A balance between its synthesis and breakdown determines the levels of ACh, which are closely correlated with the levels of ChaT and AChE [30]. SCO, a drug that blocks the action of muscarinic receptors, can cross the blood–brain barrier and mimic the memory decline symptoms seen in humans and rodents with Alzheimer’s disease and aging [31]. SCO injection inhibits ACh transmission to the post-synaptic membrane, hence increasing the activity of AChE in breaking down ACh by hydrolysis [32]. Previous in vitro studies have reported that the essential oils of *A. rugosa* inhibit AChE and butyrylcholinesterase activity, potentially improving cognitive function [33]. In this study, ARE treatment effectively reduced AChE mRNA overexpression and suppressed ChaT and mAChR1 mRNA expression. Therefore, ARE provides a neuroprotective effect via the regulation of the cholinergic system.

Due to the critical role that adult hippocampal neurogenesis plays in memory formation, neurodegenerative diseases are considered to be pathologically marked by decreased neurogenesis and neuronal integration [34]. SCO has been found to inhibit adult neurogenesis and impair the dendritic development of immature neurons in the dentate gyrus in the hippocampus [35]. However, ARE treatment effectively reverses the inhibitory effects of SCO on neurogenesis in the hippocampal dentate gyrus area.

The findings of the DPPH and NO assays, respectively, indicate that ARE has significant antioxidant and anti-inflammatory properties. These attributes most likely play a role in ARE’s neuroprotective effects against SCO-induced hippocampal damage. SCO is recognized for its ability to cause oxidative stress by promoting ROS production and reducing antioxidant defenses [36]. ROS are typically classified as neurotoxic molecules, impairing synaptic plasticity and memory function [37]. In general, gallic acid has a better DPPH inhibitory activity than the ARE. Based on the standard level of antioxidant activity proposed, the smaller the IC_50_ value, the higher the antioxidant activity, where the antioxidant capacity is very strong. Based on previous research on the antioxidant category [38], the antioxidant activity of ARE was included in the strong category because the IC_50_ value was in the range of 50–100 μg/mL. Additionally, SCO can lead to inflammatory stress by boosting the levels of pro-inflammatory cytokines [39]. The antioxidant properties of ARE assist in reducing oxidative damage, while its anti-inflammatory properties serve to decrease neuroinflammation. Both effects are crucial in protecting hippocampal neurons. Despite our findings demonstrating significant improvements in learning, memory and antioxidant properties, we acknowledge the need for further exploration into the specific antioxidant molecular mechanisms. Specifically, we did not analyze antioxidant enzymes such as superoxide dismutase, catalase and glutathione, which play essential roles in oxidative stress at the cellular level [13]. Additionally, a more detailed analysis of AChE activity would deepen our understanding of ARE’s cholinergic modulation. Future studies should evaluate these enzyme activities and explore the interactions of ARE with these enzymes to gain a comprehensive view of its neuroprotective effects. Despite this limitation, our results provide a strong foundation for further investigation into ARE as a potential cognitive enhancer.

Additionally, tilianin, acacetin and rosmarinic acid have been identified as the main compounds in *A. rugosa* [40]. Specifically, tilianin and acacetin are the predominant components in the 70% ethanol extract of *A. rugosa* [41]. Tilianin has been shown to provide neuroprotection in a rodent model of vascular dementia by acting through anti-inflammatory and anti-apoptotic pathways [42]. Additionally, acacetin has demonstrated neuroprotective properties by inhibiting oxidative damage and neuroinflammation [43,44]. These flavonoids likely contribute to the memory enhancement, antioxidant and anti-inflammatory effects of ARE observed in this study. Although this investigation did not focus on the anti-amnestic properties of individual molecules such as tilianin and acacetin, further research is needed to fully understand their neuroprotective mechanisms. These findings suggest that ARE could be a promising candidate for developing cognitive enhancers to combat memory impairments associated with neurodegenerative conditions.

## 4. Materials and Methods

### 4.1. Animals

**Figure 5:**
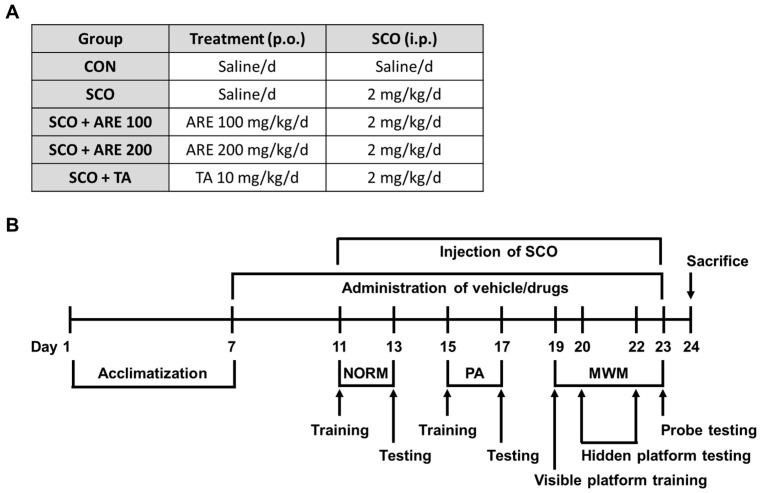
The time schedule of the experimental and treatment design. (**A**) SCO was used at 2 mg/kg. ARE was used at 100 mg/kg (ARE 100) or 200 mg/kg (ARE 200). All solutions were dissolved in saline. (**B**) The experimental schedule of testing scopolamine-induced memory deficits in the NORM, PA and MWM was used to evaluate the behavioral memory of the mice at the selected time points. SCO, scopolamine; NORM, novel object recognition memory test; PA, passive avoidance test; MWM, Morris water maze test; ARE, *Agastache rugosa* extract; TA, tacrine; p.o., per oral; i.p., intraperitoneal; CON, control.

A total of 24 male C57BL/6 mice, 22–25 g, aged 7 weeks, were purchased from Doo Yeol Biotech in Seoul, Republic of Korea. The mice were housed in a controlled environment with a temperature range of 23 °C ± 2 °C, a relative humidity of 50% ± 5%, an artificial lighting from 08:00 to 20:00 and an air exchange rate of 13–18 cycles per hour. The mice were fed a standard laboratory diet and had free access to water. The experimental and animal handling procedures were conducted in compliance with the guidelines set by the institutional care and use committee of Chonnam National University (CNU IACUC-YB-2022-27) and in accordance with the National Institute of Health’s (NIH) Guide for the Care and Use of Laboratory Animals. After 1 week of quarantine and acclimation, the mice were randomly divided into the following four groups (*n* = 6 per group; Figure 5A): control (CON; saline per oral [p.o.] daily + saline intraperitoneally [i.p.] daily); SCO (saline p.o. daily + SCO 2 mg/kg i.p. daily); ARE therapy (ARE 100 or 200 mg/kg p.o. daily + SCO 2 mg/kg i.p. daily) and TA therapy (TA 10 mg/kg p.o. daily + SCO 2 mg/kg i.p. daily). In a previous in vivo study, ARE revealed that HCl/EtOH-induced gastritis ameliorates the effect at 100 and 200 mg/kg/day doses. In this study, we determined the ARE dosage based on a previous study [19].

### 4.2. Preparation of ARE

*A. rugosa* was obtained from Jeju Island (Seongsan-eup, Seogwipo, Republic of Korea [33°27′47.5″ N, 126°54′48.9″ E, altitude 17 m]). Professor Jun-Ho Song, a plant taxonomist from Chungbuk National University in Cheongju, Chungbuk, Republic of Korea, performed the identification of *A. rugosa*. To obtain a total extract, 100 g of dried whole plant of the ground part of *A. rugosa* was added to a 1 L solution of 20% EtOH (*v*/*v*), and reflux extraction procedures were conducted for 2 h at 30 °C. Next, the extract was filtered and concentrated with a rotary evaporator (Rotavapor R-100, Buchi, Flawil, Switzerland). The concentrated extract was then lyophilized using a freeze dryer (IlshinBioBase Co., Ltd., Jeonju-si, Republic of Korea). Finally, the total extract was stored at −80 °C until use.

### 4.3. Drug Administration

The ARE was dissolved in saline and administered orally. SCO hydrobromide was acquired from Sigma-Aldrich (St. Louis, MO, USA), dissolved in saline and administered intraperitoneally. The ARE was given to the mice 1 h before behavioral testing. SCO was injected following the 30 min administration of the ARE. The experimental procedure is shown in Figure 5B.

### 4.4. Behavior Measurements

#### 4.4.1. Passive Avoidance Test

The PA followed the protocol established in our previous study [34], utilizing a PA device (Ugo Basile, Gemonio, Italy). The behavior test consisted of two phases—training and testing—with a 24 h interval between them. During the training session, the mice were given 60 s to explore the bright chamber before the trap door was accessible. Once they entered the dark chamber, the trap door closed and the mice received a foot shock of 0.5 mA for 2 s. After 20 s in the dark chamber, the mice were brought back to their original cage. During the testing session, the mice were placed in the bright chamber once more and the trap door was opened. The duration of time spent entering the dark chamber was recorded as the escape latency (s). All mice were subjected to a time limit of 540 s.

#### 4.4.2. Novel Object Recognition Memory Test

The NORM followed the protocol established in our previous study [20]. Mice were individually acclimated to an acrylic chamber (42 × 28 × 20 cm) for three consecutive days. For this behavior test, immobile plastic objects with varying shapes and heights of 3.5 cm were utilized. The behavior test consisted of two phases—training and testing—with a 24 h interval between them. During the training session, each mouse was shown two randomly selected items of different shapes for 10 min. During the testing session, a second batch of items (consisting of one object that had been previously presented and another object that was new) was presented to the trained mice. An interaction was recorded when each mouse exhibited olfactory investigation or tactile exploration of an object, or approached an object within less than 1 cm from their nose tip. The preference rate for each object was calculated by dividing the number of interactions with that object by the total number of interactions with either object.

#### 4.4.3. Morris Water Maze Test

The MWM followed the protocol established in our previous study [45]. The MWM apparatus consisted of a circular tank 100 cm in diameter and 50 cm in height, surrounded by various visual indicators. The tank was filled with water, which was kept at 25 °C, to a height of 30 cm. To make the water opaque, a non-toxic, washable white paint was used. Additionally, the tank was divided into four virtual quadrants of the same size. A white platform, measuring 10 cm in diameter and 25 cm in height, was positioned at the center of one of the quadrants. The behavior test consisted of three phases—visible platform training (1 day, four trials per day), probe testing (3 days, four trials per day) and hidden platform testing (1 day, once per day). During the visible platform training session, each mouse received individual training in a circular pool. A platform for escaping was positioned in the middle of a specific quadrant of the pool and marked by a flag 5 cm in height. The mice were trained for 1 day, with four trials at 1 h intervals. Each trial had a duration of 60 s, unless the mouse successfully reached the platform. If the mouse failed to reach the platform within 60 s, it was manually guided to the platform. The mouse was given a 30 s time limit on the platform, regardless of whether it successfully reached the platform within 60 s. During the hidden platform testing session, the platform was submerged in the opaque water without any indication of its location. The platform was positioned in a fixed location and the beginning locations were pseudo-randomly adjusted between trials. During the probe testing session, the platform was removed from its fixed location and the mice were allowed to swim in the pool for 1 min. Swimming velocity was measured throughout the first visible platform training session. During the probing testing session, we measured the duration of time spent in the quadrant and the frequency of crossings over the concealed platform location. The data collection process utilized the SMART video tracking system (Panlab, Barcelona, Spain).

#### 4.4.4. Immunohistochemistry

The immunohistochemistry followed the protocol established in our previous study [15]. The brain hemispheres, which had been fixed with 4% paraformaldehyde, were sectioned longitudinally at a 4 μm thickness. Next, immunohistochemistry analysis was performed using rabbit antibodies to detect the KI67 antigen (1:500; Acris Antibodies GmbH, Hiddenhausen, Germany) and doublecortin antigen (1:500; Cell Signaling Technology, Beverly, MA, USA). Subsequently, the sections were incubated with biotinylated goat anti-rabbit IgG (Vector ABC Elite Kit; Vector Laboratories, Burlingame, CA, USA) and then with an avidin–biotin complex (Vector ABC Elite Kit). The peroxidase reaction was detected using the diaminobenzidine substrate from a DAB kit (SK-4100; Vector Laboratories). The sections were then counterstained with hematoxylin and were mounted. The Motic EasyScan Digital Slide Scanner (Motic, Hong Kong, China) digitalized the stained section. The staining intensity within the hippocampus was quantified using Image J (version 1.53f51, NIH, Bethesda, MD, USA).

### 4.5. Reverse Transcription Quantitative Polymerase Chain Reaction Analysis

The RNA extraction, complementary DNA (cDNA) synthesis and reverse transcription quantitative real-time polymerase chain reaction (RT-qPCR) procedures were conducted according to previously established protocols [15]. Initially, RNA was extracted from spinal cord tissue using the TRIzol reagent (79306; Qiagen, Hilden, Germany), according to the manufacturer’s guidelines. Next, the cDNA was synthesized using the SuperiorScript III cDNA synthesis kit (EZ405; Enzynomics, Daejeon, Republic of Korea). The cDNA was then diluted using RNase-free water to a final concentration of 8 ng/µL and was stored at −80 °C for preservation. Afterward, the RT-qPCR analysis was performed using the TOPreal SYBR Green qPCR PreMix (RT500M; Enzynomics) and the CFX Opus 96 instrument (Bio-Rad Laboratories, Hercules, CA, USA) following the manufacturer’s guidelines. The annealing temperature for the reaction ranged from 55 °C to 60 °C and the software automatically generated amplification curves and calculated the threshold cycle values. The GAPDH reference gene was used to standardize all the gathered data. The mean related values were compared with the normal group using the 2^−∆∆CT^ method [46]. The mouse primers used in the analysis were as follows: AChE _(NM_NM_001290010.1) forward 5′-AGC AAT ATG TGA GCC TGA ACC TGA AG-3′ and reverse 5′-CTC CGC CTC GTC CAG AGT ATC G-3′; ChaT_(NM_009891.2) forward 5′-ATT GGG TCT CTG AAT ACT GGC TGA ATG-3′ and reverse 5′-TGG TCA TTG GTG TCT TGG AAG TGC-3′; mAChR1_(NM_001112697) forward 5′-AGT GGC ATT CAT CGG GAT CA-3′ and reverse 5′-CTT GAG CTC TGT GTT GAC CTT GA-3′; GAPDH_(NM_008084.2) forward 5′-TCC ATG ACA ACT TTG GCA TT-3′ and reverse 5′-GTT GCT GTT GAA GTC GCA GG-3′.

### 4.6. DPPH Radical Scavenging Assay

Free radical scavenging activity was assessed using a modified version of the method proposed in a previous study [47]. In brief, 100 μL of the ARE (5, 10, 20, 100 and 500 μg/mL) or standard compounds (gallic acid: 5, 10, 20 and 100 μM) dissolved in 50% ethanol were added to 100 μL of 0.2 mM DPPH (Fisher Scientific, Leicestershire, UK) solution, reaching a final concentration of 0.1 mM DPPH. Each reaction mixture was shaken for 5 s and left to stand for 30 min in the dark at room temperature. The absorbance of each mixture was measured at 515 nm against a blank (50% ethanol) using a SpectraMax i3x Microplate spectrophotometer (Molecular Devices, Downingtown, PA, USA). The inhibition rate of DPPH radicals was calculated as follows:

DPPH inhibition rate (%) = (1 − [A_sample_ − A_blank_]/[A_control_ − A_blank_]) × 100

### 4.7. Determination of NO Generation in RAW 264.7 Cells

NO generation was assessed using a modified version of the method proposed in a previous study [48]. The RAW 264.7 macrophage cell line was obtained from the American Type Culture Collection (ATCC, Rockville, MD, USA). Macrophages were cultured in Dulbecco’s modified eagle medium supplemented with 10% fetal bovine serum and 1% penicillin (100 units/mL) or streptomycin (100 µg/mL) at 37 °C in 5% CO_2_. The media concentrations of NO were examined using the Griess reagent kit (Promega, Madison, WI, USA). RAW 264.7 cells (1.0 × 10^4^/well) were precultured in 96-well plates before treatment with LPS (1 μg/mL) at various ARE concentrations (25, 50, 100 and 200 μg/mL) for 24 h. NO production was measured at 540 nm and was quantified using a sodium nitrite standard curve.

### 4.8. Statistical Analysis

The results are expressed as the mean ± standard error of the mean (SEM). All statistical analyses were performed using GraphPad Prism 9.0 (GraphPad, San Diego, CA, USA). A one-way analysis of variance followed by Tukey’s multiple comparison test was used to prove statistically significant differences between groups. Statistical significance was defined as *p* < 0.05.

## Data Availability

The datasets generated and/or analyzed during the current study are available upon request from the corresponding author.
